# Remdesivir associated sinus bradycardia in patients with COVID-19: A prospective longitudinal study 

**DOI:** 10.3389/fphar.2022.1107198

**Published:** 2023-01-17

**Authors:** Maryam Hajimoradi, Babak Sharif Kashani, Farzaneh Dastan, Sina Aghdasi, Atefeh Abedini, Farah Naghashzadeh, Arezoo Mohamadifar, Mohammad Sadegh Keshmiri, Sima Noorali, Somayeh Lookzadeh, Niloufar Alizadeh, Mohammad Amin Siri, Mohammadali Tavasolpanahi, Yazdan Abdolmohammadi, Masoud Shafaghi, Zahra Sadat Rouhani, Shadi Shafaghi

**Affiliations:** ^1^ Lung Transplantation Research Center, National Research Institute of Tuberculosis and Lung Diseases (NRITLD), Shahid Beheshti University of Medical Sciences, Tehran, Iran; ^2^ Chronic Respiratory Diseases Research Center, National Research Institute of Tuberculosis and Lung Diseases (NRITLD), Shahid Beheshti University of Medical Sciences, Tehran, Iran; ^3^ Department of Clinical Pharmacy, School of Pharmacy, Shahid Beheshti University of Medical Sciences, Tehran, Iran; ^4^ Department of Biostatistics, National Research Institute of Tuberculosis and Lung Disease (NRITLD), Shahid Beheshti University of Medical Sciences, Tehran, Iran; ^5^ Tracheal Diseases Research Center, National Research Institute of Tuberculosis and Lung Diseases, Shahid Beheshti University of Medical Sciences, Tehran, Iran; ^6^ Strategic Planning and Executive Office Manager of International Federation of Inventors' Associations-IFIA, Geneva, Switzerland

**Keywords:** remdesivir, bradycardia, COVID-19, arrhythmia, ECG, cardiotoxicity, SARS-CoV-2

## Abstract

**Background:** Remdesivir is effective against SARS-Cov-2 with little evidence of its adverse effect on the cardiac system. The aim of the present study is investigating the incidence of bradycardia in COVID-19 patients treated with Remdesivir.

**Methods:** This prospective longitudinal study was conducted in a tertiary center on COVID-19 patients for Remdesivir therapy. The objectives were to investigate the incidence of sinus bradycardia, and also the association between their demographics, underlying diseases, and the disease severity with developing bradycardia in COVID-19 patients treated with Remdesivir.

**Results:** Of 177 patients, 44% were male. The mean (±standard deviation) age of patients was 49.79 ± 15.16 years old. Also, 33% were hospitalized due to more severe symptoms. Oxygen support was required for all hospitalized subjects. A total of 40% of the patients had comorbidities, with the most common comorbidity being hypertension. The overall incidence of bradycardia (heart rate<60 bpm) in patients receiving Remdesivir was 27%, of whom 70% had extreme bradycardia (heart rate <50 bpm). There was also a statistically significant reduction in heart rate after five doses of Remdesivir compared to the baseline heart rates. In the multivariable model, none of the covariates including age above 60 years, female sex, CRP>50 mg/L, O2 saturation<90%, underlying cardiovascular disease, hypertension and diabetes mellitus, and beta-blockers were associated with Remdesivir-induced bradycardia. No association was found between the COVID-19 severity indicators and bradycardia.

**Conclusion:** As sinus bradycardia is a prevalent adverse cardiac effect of Remdesivir, it is recommended that all COVID-19 patients receiving Remdesivir, be evaluated for heart rate based on examination; and in the case of bradyarrhythmia, cardiac monitoring should be performed during administration to prevent adverse drug reactions.

## 1 Introduction

The Coronavirus disease 2019 (COVID-19) first appeared in Wuhan, China, in December 2019 and was stated a pandemic by the world health organization (WHO) in March 2020. It rapidly spread around the world and has accounted for millions of global deaths since then. (Chen et al., 2020; Ganesh et al., 2021). The disease is caused by the severe acute respiratory syndrome coronavirus-2 (SARS-CoV-2), an ribonucleic acid (RNA) virus from *Corona* Viridae family. The infection causes respiratory illness and varies widely in severity from asymptomatic or mild infection to severe pneumonia and subsequent fatal complications, including acute respiratory distress syndrome (ARDS), multiple organ failure, and death. (Grein et al., 2020; Zeng et al., 2021).

Among the antiviral drugs introduced and tested for the treatment of COVID-19, Remdesivir has been particularly used to treat the infection and long-COVID syndrome (Jacinto et al., 2021) during the pandemic after demonstrating its *in-vivo* and *in-vitro* inhibitory effects against SARS-CoV-2. (Beigel et al., 2020; Gordon et al., 2020; Gubitosa et al., 2020).

Remdesivir is a nucleotide analog that implicates in viral RNA and inhibits RNA polymerase and viral replication in a wide spectrum of viruses, including SARS-CoV-2, and is potently active in primary human epithelial cells in lung airways. (Gubitosa et al., 2020; Gupta et al., 2020; Gottlieb et al., 2022).

Various studies have indicated its inhibitory effect against the SARS-Cov-2, and it has been approved as an efficient antiviral treatment for hospitalized SARS-Cov-2 patients with moderate to severe infection in all variants of concern. (Beigel et al., 2020; Goldman et al., 2020; Grein et al., 2020; Pasquini et al., 2020; Wang et al., 2020; Pallotto et al., 2021a; Barkas et al., 2021; Brunetti et al., 2021; Gottlieb et al., 2022). A recent randomized controlled trial on non-hospitalized patients infected with COVID-19 who were at higher risk of disease progression showed that Remdesivir treatment reduced the risk of hospitalization and death by 87% compared to placebo. (Gottlieb et al., 2022).

Although its beneficial role in the treatment of COVID-19 has been valued and well described in the literature, evidence on its adverse effect (ADR), especially on the cardiovascular system, is scarce, and the available studies are mainly limited to hepatic, renal, and dermal adverse drug reactions of the drug. (Sarkar et al., 2020; Pallotto et al., 2021a; Pallotto et al., 2021b; Kow et al., 2021). Bradycardia, hypotension, QT interval prolongation, atrial fibrillation, and even cardiac arrest are among the most frequently reported cardiovascular complications attributed to Remdesivir in the literature. (Beigel et al., 2020; Grein et al., 2020; Gupta et al., 2020; Wang et al., 2020). Two potential mechanisms have been proposed for these adverse cardiac effect. First, the Remdesivir active metabolite resembles adenosine triphosphate (ATP). Adenosine may inhibit sinus node automaticity and atrioventricular (AV) node conduction by its chronotropic and dromotropic effects and transiently increases the central vagal tonicity in the heart and also the myocardial repolarization time. These effects may lead to arrhythmias, sinus bradycardia, corrected QT interval (QTc) prolongation and AV node blockage, as have been recently described in the literature. The second mechanism is the Remdesivir affinity to human mitochondrial RNA polymerase, which may possibly result in mitochondrial cardiomyocyte dysfunction and toxicity. (Kumar et al., 2021a; Ching and Lee, 2021; Day et al., 2021; Jacinto et al., 2021; Sanchez-Codez et al., 2021; Touafchia et al., 2021).

Currently, there is limited data on the cardiac adverse effect of Remdesivir except for a few case reports and case series. (Gubitosa et al., 2020; Gupta et al., 2020; Barkas et al., 2021; Ching and Lee, 2021; Day et al., 2021; Jacinto et al., 2021; Selvaraj et al., 2021).

Further comprehensive studies are required to clarify the exact association between Remdesivir and adverse cardiac effects that may lead to bradycardia and other cardiac complications in COVID-19 patients receiving this medication. The present study investigates the incidence of bradycardia in SARS-Cov-2 patients who received Remdesivir and examines the effect of demographic characteristics, underlying risk factors, and the infection severity on developing sinus bradycardia as the most prevalent cardiac complication of Remdesivir. (Lucijanic and Bistrovic, 2022). The results could provide a foundation for future precautions in treating COVID-19 patients receiving Remdesivir.

## 2 Materials and methods

### 2.1 Study population

This prospective longitudinal study was conducted using data from patients admitted to Dr. Masih Daneshvari hospital -a tertiary care center for lung diseases-in Tehran, Iran, from 19 August 2021, to 7 November 2021. The patients were randomly selected from daily systemic lists of registered patients with a COVID-19 diagnosis. The inclusion criteria were 1) aged 18 years or older, 2) a confirmed diagnosis of COVID-19 infection according to positive polymerase chain reaction (PCR) test results or chest computed tomography (CT) scan findings compatible with COVID-19 diagnosis, and 3) indication for receiving Remdesivir (Rezaei et al., 2021; Mirenayat et al., 2022). (Coronavirus Disease 2019, 2021). The exclusion criteria were 1) having rhythms other than sinus at baseline electrocardiogram (ECG), 2) a heart rate (HR) < 60 beats per minute (bpm) at baseline, and 3) using a cardiac pacemaker or cardiac resynchronization therapy (CRT) device and implantable-cardioverter defibrillator (ICD).

The priory sample size was calculated 166 using the formula $n=p\left(1-p\right){\left(\frac{z_{1-\alpha /2\;}+z_{1-\beta }}{p-p_{0}}\right)}^{2}$ . The predicted incidence of bradycardia with Remdesivir P) was considered 20% and the P_0_ calculated 28.7% based on the kumar et al. study (Kumar et al., 2021a). The power considered 80% and the type 1 error considered 5%. Initially, data was collected from 188 patients as it was predicted some patients probably miss follow-up sessions and finally data of 177 patients who met the criteria and completed follow-up sessions was analyzed.

### 2.2 Data collection and follow-up

Data on patients’ demographic information, medical history, drug history, clinical condition, therapeutic management, laboratory values, and oxygen-support requirements were collected *via* an assessment form by clinicians (Baghaei et al., 2020). Patients’ age, sex, comorbidities (e.g., diabetes mellitus, hypertension, and cardiovascular diseases), basal laboratory findings representative of infection severity including D-Dimer, CRP, absolute lymphocyte count (ALC), oxygen therapy requirement and O2 saturation, temperature, potassium, sodium, BUN, Cr level, outpatient or inpatient status, and using Tocilizumab, beta-blockers, and anti-arrhythmic drugs were variables included in the multivariable analysis. All vital sign measurements were performed immediately before and after Remdesivir administration and baseline ECG was performed for all patients enrolled the study before any therapy initiation. All patients underwent five sessions of Remdesivir administration, including 100 mg Remdesivir daily following a 200 mg intravenous loading dose. Dexamethasone and venous thromboembolism prophylaxis were also administered to all patients with different dosages according to the disease severity. The heart rates of patients were examined by a pulse oximeter as soon as Remdesivir administration was finished in each session. It was reconfirmed by a second measurement and the mean of two measurements were obtained. The Heart rate below 60 bpm was considered as bradycardia and the heart rate below 50 bpm was considered as extreme bradycardia. Second ECG was conducted if bradycardia detected on examination to determine the cardiac rhythm of patients in each session. A final ECG was performed for all patients in the study after the fifth dose of Remdesivir. Characteristics of baseline and final electrocardiograms were measured and reported by two cardiologists. The characteristics of baseline and final ECGs including the ventricular rate, PR duration, QRS width, QT interval duration, and QTc were extracted by two cardiologists and the baseline and final ECG characteristics were compared using the Wilcoxon rank test (*p*-value = .05). Severe bradycardia was defined as heart rate <50 bpm. (Drumheller et al., 2022). QTc was calculated through Bazett’s formula $\left(QTc=\frac{QT}{\sqrt{RR}}\;\right)$ (Bazett, 1997). QTc>460 ms in women and QTc>440 ms in men were considered as the prolongation of the QTc interval. Absolute QTc≥500 ms (millisecond) was considered as extreme QTc prolongation. (Russo et al., 2020). Patients who developed bradycardia were followed 2 weeks after drug cessation for their heart rate to investigate if this was a temporary effect.

### 2.3 Ethical approval and consent to participate

An informed consent form was reviewed and signed by all patients before participation. The study obtained the approval of the Iran National Committee for Ethics in Biomedical Research and followed the national standards for performing Medical Research in Iran (Ethic code: IR. SBMU.NRITLD.REC.1400.050, approval date: 2021-09-26), and the ethical guidelines outlined in the 1975 Helsinki Declaration.

### 2.4 Aims and objectives

The primary objective of the present study was to investigate the incidence of sinus bradycardia in COVID-19 patients receiving Remdesivir treatment. The secondary objective was to investigate the association of patients’ underlying risk factors and diseases and also the severity of the COVID-19 infection with developing bradycardia in these patients.

### 2.5 Statistical analysis

The Kolmogorov-Smirnov and Shapiro-Wilk normality tests were used to examine the distribution of variables. Quantitative data were described by the median and interquartile range (IQR). For qualitative data, the frequency and percentage were calculated. For comparing means (or medians) between two groups, the T-test or Mann-Whitney U test were used for quantitative variables. To determine if the difference between observed and expected data is due to chance or due to a relationship between the qualitative variables, we used chi-square (or exact fisher tests) and Odds ratio for measuring (quantify) the strength (size) of association between them. Friedman and Wilcoxon signed-rank tests were used to investigate the changes within the repeated measured variables. To explain the relationship between bradycardia and underlying factors logistic regression analysis was assessed through multivariable analyses. The data was analyzed using statistical package for the social sciences (SPSS) software version 22, and a *p*-value below .05 was considered statistically significant in all analyses.

## 3 Results

### 3.1 Patients’ characteristics

Of the total 188 patients, two were excluded due to arrhythmia at baseline ECG, and nine were excluded because of missing follow-up information or final ECG or incomplete courses of Remdesivir therapy. Overall, the data of 177 patients, of which 44% were male, were analyzed. The baseline clinical characteristics of the patients are shown in Tables 1, 2. The Mean ± standard deviation (SD) age of patients was 49.79 ± 15.16 years (minimum 19, maximum 88). Of the total patients, 33% were hospitalized due to more severe symptoms, of which 98% were admitted to the COVID-19 ward and 2% to the intensive care unit (ICU). The rest of the patients (67%) were outpatients with less severe symptoms who were admitted to the hospital to receive Remdesivir and were discharged after each session of drug administration (Rezaei et al., 2021; Mirenayat et al., 2022). The most common symptoms at admission were cough (80%) and dyspnea (56%). Oxygen support was required for 33% of patients, of whom 18% were supplied with oxygen through a high flow nasal cannula (HFNC), 13% through a non-rebreather face mask, and 2% through bi-level positive airway pressure (BiPAP). None of the studied patients were intubated. A total of 40% of patients had comorbidities, with the most common comorbidity being hypertension, with a prevalence of 14%. Also, 13% of patients were diabetic, and 9% had an underlying cardiovascular disease. Patients’ medication history at admission showed that 12.4% used beta-blockers. The median of onset of symptoms to admission for patients was 8.13 ± 3.76 days.

**TABLE 1:** Comparing medians of quantitative variables between two groups of patients (heart rate<60 and heart rate≥60) using Mann-Whitney U test.

Characteristic	Heart Rate<60 bpm								*p*-value
	No				Yes				
	Mean	Standard Deviation	Median	IQR	Mean	Standard Deviation	Median	IQR	
Age, year	50	15	47	21	49	14	48	21	.754
BMI	29.0	7.6	26.6	7.0	26.1	3.9	26.0	6.2	.227
Temperature, ˚C	36.669	.638	36.500	.900	36.627	.528	36.700	.550	.698
BP(systolic)	118	9	120	10	117	12	118	15	.516
BP(diastolic)	74.78	8.89	75.00	10.00	74.40	8.36	72.50	10.00	.657
HR, bpm	97	17	96	21	97	14	98	16	.826
O2_sat, %	92	5	93	5	91	5	92	8	.253
WBC	6462.30	3320.10	5665.00	3160.00	7308.50	3436.51	6150.00	3900.00	.100
Hb, g/dL)	13.501	1.907	13.650	2.400	13.704	1.382	13.500	2.050	.972
Plt	204	86	185	107	207	81	186	81	.763
Lymph	1,203	549	1,054	576	1,185	463	1,079	536	.833
Neut	4,779	2,931	3815	2,872	5840	3463	4,760	4,246	.071
K,mmol/L	4.024	.417	4.000	.600	4.112	.395	4.100	.500	.274
D_Dimer, ng/mL	1778.833	1,258.294	1,363.500	1,437.000	1,348.400	882.836	1,309.000	1,078.000	.855
Troponin, ng/mL	.058	.052	.040	.075	.020	.000	.020	.000	.273
LDH, IU/L	630.560	198.785	653.000	332.000	660.900	261.091	605.000	397.000	.841
Bs, mg/dL	180.118	90.376	135.000	121.000	173.250	104.460	121.500	100.000	.705
BUN, mg/dL	37.452	24.871	31.500	15.000	37.250	17.712	31.000	16.000	.689
Cr, mg/dL	1.059	.610	1.000	.210	1.016	.234	1.000	.400	.935
AST, U/L	39	24	33	24	48	38	32	34	.348
ALT, U/L	44	40	30	33	51	43	35	27	.244
ALK.ph, U/L	174	68	160	61	170	73	163	48	.737
Ca, mg/dL	9.113	1.527	9.250	1.000	9.617	.483	9.700	.900	.529
Mg, mg/dL	2.287	.338	2.300	.600	2.225	.560	2.150	.650	.497
PT, seconds	12.837	2.800	12.250	.700	16.655	23.410	12.200	1.200	.447
INR	1.080	.273	1.020	.070	1.056	.073	1.010	.105	.776
ESR, mm/hr	47.667	29.200	51.500	53.000	47.000	3.606	48.000	7.000	1.000
CRP,mg/L	21	19	15	37	24	18	18	33	.412
CPK, mcg/L	150	225	67	58	141	127	89	141	.427

IQR**:** Interquartile Range**,** BMI**:** Body mass index**,** BP**:** Blood Pressure**,** HR: Heart Rate**,** O2_sat: Oxygen saturation,WBC: White Blood Cell**,** Hb: Hemoglobin**,** Plt: Platelet**,** Lymph: Lymphocyte**,** Neut: Neutrophil**,** K: Potassium, LDH: Lactate dehydrogenase**,** BS: Blood Sugar, BUN: Blood Urea Nitrogen**,** Cr: Creatinine**,** AST: Aspartate transaminase**,** ALT: Alanine transaminase**,** ALK. ph: Alkaline phosphatase**,** Ca: Calcium**,** Mg: Magnesium**,** PT: Prothrombin Time**,** INR: International Normalized Ratio**,** ESR: Erythrocyte sedimentation rate**,** CRP: C-Reactive Protein**,** CPK: Creatine Phosphokinase**,** *p*-value<.05 was considered statistically significant.

*p*-value<.05 was considered statistically significant.

**TABLE 2:** Evaluating the association between qualitative variables and bradycardia using chi-square or fisher’s exact test* and Odds ratio (OR) for measuring the strength (size) of association between them.

Characteristics			Heart rate<60		Total	*p*-Value	OR
			No	Yes			
Sex	Male	Count	58	19	77	.495	1.2
		% of Total	33.0%	10.8%	43.8%		
	Female	Count	70	29	99		
		% of Total	39.8%	16.5%	56.3%		
Age (Years)	Under 60	Count	92	37	129	.534	0.7
		% of Total	52.6%	21.1%	73.7%		
	60 and above	Count	35	11	46		
		% of Total	20.0%	6.3%	26.3%		
		% of Total	71.8%	26.4%	98.3%		
	Critical	Count	3	0	3		
		% of Total	1.7%	.0%	1.7%		
Oxygen therapy	Nasal cannula	Count	20	11	31	.491*	
		% of Total	36.4%	20.0%	56.4%		
	Reservoir mask	Count	13	7	20		
		% of Total	23.6%	12.7%	36.4%		
	BiPAP	Count	4	0	4		
		% of Total	7.3%	.0%	7.3%		
Admission	Outpatient	Count	92	27	119	.058	1.9
		% of Total	52.0%	15.3%	67.2%		
	Inpatient	Count	37	21	58		
		% of Total	20.9%	11.9%	32.8%		
Need of oxygen support	No	Count	89	28	117	.190	1.6
		% of Total	51.1%	16.1%	67.2%		
	Yes	Count	38	19	57		
		% of Total	21.8%	10.9%	32.8%		
		% of Total	67.5%	28.4%	95.9%		
Comorbidities	Yes	Count	50	19	69	.848	0.9
		% of Total	29.1%	11.0%	40.1%		
	No	Count	76	27	103		
		% of Total	44.2%	15.7%	59.9%		
Hypertension	No	Count	107	41	148	.481	0.7
		% of Total	62.2%	23.8%	86.0%		
	Yes	Count	19	5	24		
		% of Total	11.0%	2.9%	14.0%		
Diabetes	No	Count	110	39	149	.667	1.2
		% of Total	64.0%	22.7%	86.6%		
	Yes	Count	16	7	23		
		% of Total	9.3%	4.1%	13.4%		
Cardiovascular disease	No	Count	119	38	157	.028*	3.6
		% of Total	69.2%	22.1%	91.3%		
	Yes	Count	7	8	15		
		% of Total	4.1%	4.7%	8.7%		
Hypercholesterolemia	Yes	Count	1	2	3	.175*	0.2
		% of Total	.6%	1.2%	1.7%		
	No	Count	125	44	169		
		% of Total	72.7%	25.6%	98.3%		
Thyroid disease	Yes	Count	3	0	3	.565*	
		% of Total	1.7%	.0%	1.7%		
	No	Count	123	46	169		
		% of Total	71.5%	26.7%	98.3%		
Chronic kidney disease	Yes	Count	2	0	2	.999*	
		% of Total	1.2%	.0%	1.2%		
	No	Count	124	46	170		
		% of Total	72.1%	26.7%	98.8%		
		% of Total	3.1%	1.3%	4.4%		
Anti-arrhythmia	No	Count	122	41	163	.056*	8.9
		% of Total	73.1%	24.6%	97.6%		
	Yes	Count	1	3	4		
		% of Total	.6%	1.8%	2.4%		
		% of Total	68.9%	25.1%	94.0%		
Azithromycin	No	Count	127	47	174	.999*	1.3
		% of Total	71.8%	26.6%	98.3%		
	Yes	Count	2	1	3		
		% of Total	1.1%	.6%	1.7%		
Furosemide	No	Count	113	40	153	.763	1.1
		% of Total	67.7%	24.0%	91.6%		
	Yes	Count	10	4	14		
		% of Total	6.0%	2.4%	8.4%		
Beta-blocker	No	Count	115	40	155	.297	1.6
		% of Total	65.0%	22.6%	87.6%		
	Yes	Count	14	8	22		
		% of Total	7.9%	4.5%	12.4%		

*p*-value<.05 was considered statistically significant. Note: chi-square test used unless otherwise noted. *fisher’s exact test used.

**FIGURE 1:**
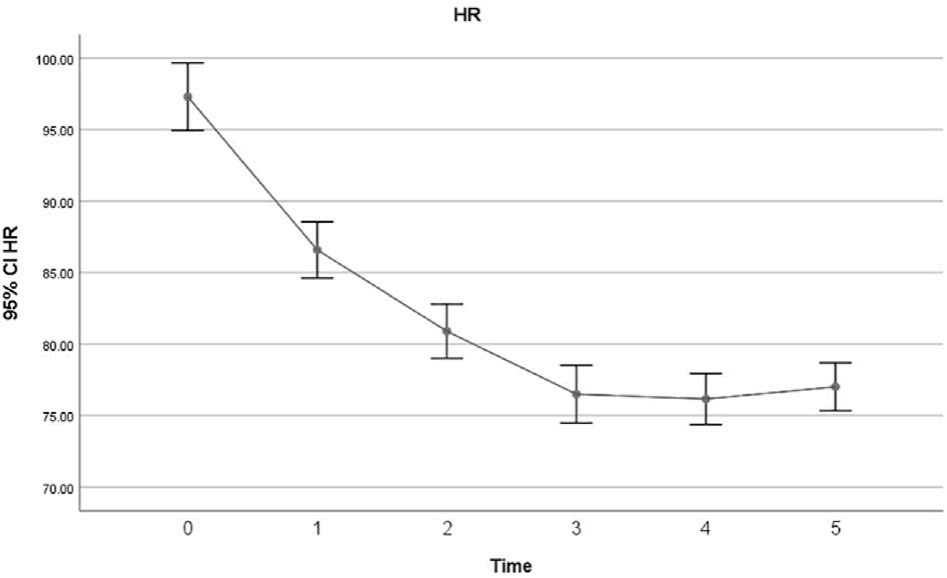
time (second, s), heart rate (beats per minute, bpm).

The overall incidence of bradycardia (heart rate<60) in patients receiving Remdesivir was 27%, and 19% of patients developed extreme bradycardia (heart rate <50 bpm). None of the patients developed bradycardia in the first session of Remdesivir treatment. 2% developed bradycardia in the second session, 7% in the third session, 6% in the fourth session and 21% in the last session. The mean heart rates of patients in each session of Remdesivir treatment are shown in Figure 1. All except one patient with HR 25 bpm had asymptomatic bradycardia. Sinus bradycardia lasted up to 2 weeks after Remdesivir discontinuation. There were no significant differences in primary clinical characteristics in the bradycardia patients and others, and developing bradycardia did not affect the clinical outcome of patients in the study. Nevertheless, the CRP levels were not suggestive of developing bradycardia in patients in the present study (*p*-value = .41). All studied patients had a favorable prognosis regardless of developing bradycardia, and no case of mortality or intubation during hospitalization was observed.

A change of 5 mm Hg was observed in the mean diastolic blood pressure after Remdesivir administration, which was statistically significant (*p*-value = .001). However, changes in the mean systolic blood pressure were not significant (*p*-value = .058)

There was no association between any infection severity indicator and bradycardia. Although an underlying cardiovascular disease and Tocilizumab had a correlation with bradycardia in multivariable analysis, it was not confirmed in the multivariate logistic model (Table 3). The covariates of age above 60, female sex, c-reactive protein (CRP) > 50 mg/L, O2 saturation <90%, underlying cardiovascular disease, hypertension (HTN) and diabetes mellitus, and beta-blockers were used as inputs in the multivariable regression analysis model. The results showed that none of these factors were associated with bradycardia in COVID-19 patients receiving Remdesivir (Table 3)

**TABLE 3:** Univariable and multivariable logistic regression model of predictor variables for bradycardia.

Characteristics	Univariable analysis		Multivariable analysis	
	OR (95% CI)	P	OR (95% CI)	P
Age>60	.994 (.972–1.016)	.598	2.237 (.679–7.369)	.185
Female gender	.791 (.403–1.553)	.495	.825 (.359–1.895)	.650
D-dimer	1.000 (.998–1.001)	.498		
Lymphocyte	1.000 (.999–1.001)	.850		
O2 sat<90%	.965 (.903–1.032)	.296	.459 (.147–1.435)	.181
CRP>50	1.007 (.987–1.027)	.525	2.022 (.364–11.235)	.421
Beta-blocker	.609 (.238–1.559)	.301	.115 (.010–1.321)	.083
Temperature	.890 (.509–1.555)	.683		
CVD	.279 (.095–.821)	.020	.589 (.097–3.571)	.564
Cr	.817 (.329–2.034)	.665		
BUN	1.000 (.985–1.015)	.960		
Diabetes	.810 (.310–2.117)	.668	.986 (.238–4.085)	.985
Hypertension	1.456 (.510–4.156)	.483	4.349 (.486–38.895)	.188
Tocilizumab	2.583 (1.106–6.034)	.028		

CVD, cardiovascular diseases; CRP, C-reactive protein; Cr, creatinine; BUN, blood urea nitrogen.

### 3.2 Electrocardiographic characteristics

The characteristics of baseline and final ECGs are summarized in Table 3. The baseline and final ECG characteristics were compared using the Wilcoxon rank test (*p*-value = .05), and the changes in ventricular rate, QT interval, and QTc interval were statistically significant (Table 4). There was also a statistically significant reduction in heart rate after five doses of Remdesivir compared to the baseline heart rates (87.43 ± 15.52 at baseline vs. 67.62 ± 14.81) (*p* < .001). Mean heart rate changes (±95% confidence interval (CI)) after each Remdesivir administrations are shown in Figure 1. As shown in Table 5, ECG parameters like ventricular rate, QT, and QTc interval durations changed significantly after Remdesivir administration. In the present study, the mean QTc interval duration shortened significantly after the fifth dose of Remdesivir compared to baseline (reduced 6 ms, *p*-value = .026). Of all patients, 9.1% had QTc interval prolongation prior to Remdesivir administration, and 6.7% developed QTc interval prolongation afterward. Three patients (two women and a man) aged 37 to 42 developed extreme QTc prolongation (QTc>500 ms), but none of the patients developed an arrhythmia, including torsades de pointes and atrial fibrillation.

**TABLE 4:** Ventricular rate changes before and after Remdesivir administration.

Rate	Mean	Std. Deviation	Minimum	Maximum	Percentiles		
					25th	50th (Median)	75th
Basal	87.43	15.522	60	130	75.00	88.00	100.00
Final	67.62	14.814	25	115	60.00	65.00	75.00

**TABLE 5:** ECG characteristics before and after Remdesivir administration.

Characteristics	Basal ECG	Final ECG	*p*-Value
Ventricular rate (bpm)	87.43 ± 15.522	67.62 ± 14.814	.000
PR segment duration (ms)	144.17 ± 31.059	149.59 ± 35.469	.080
QT interval duration (ms)	335.78 ± 44.891	381.98 ± 82.232	.000
QTc interval duration (ms)	400.35 ± 39.429	394.52 ± 54.413	.026
QRS width (ms)	79.94 ± 39.209	78.65 ± 29.545	.281

Abbreviations: bpm, beats per minute; ECG, electrocardiographic; ms, millisecond*p < .05 considered statistically significant, using Wilcoxon signed test for paired samples.

## 4 Discussion

The present study evaluated the incidence of sinus bradycardia, the most frequent cardiovascular adverse drug reaction of Remdesivir (Lucijanic and Bistrovic, 2022), in patients infected with COVID-19 and also the association between patients’ demographic characteristics, clinical conditions, and the severity of COVID-19 and developing bradycardia.

The prevalence of bradycardia following Remdesivir administration varies widely based on the literature review, from only 3.6% to up to 60%. This wide diversity may be due to the differences in patients’ demographic characteristics, comorbidities and risk factors, medication history, and severity of COVID-19 infection, different study designs and selection bias. Based on the data in the present study, the overall incidence of bradycardia was 27% with 19% HR < 50 bpm, which further supports previous findings on the association between Remdesivir and bradycardia. Palloto et al. found 60% bradycardia incidence after Remdesivir administration *versus* 23% in the control group. However, their sample size was small (46 patients) and included only 20 patients in the Remdesivir group. They found that the age >65 years and Remdesivir were associated with bradycardia. (Pallotto et al., 2021a). In another retrospective study on 141 patients, the incidence of bradycardia in Remdesivir group was significantly higher (46.8% compared to 27.8% in the control group (OR = 2.15)). (Pallotto et al., 2021b). In a recent study of 180 patients with COVID-19 infection who received Remdesivir, 28.7% developed bradycardia, similar to the incidence of bradycardia in this study. (Kumar et al., 2021a). In the study by Toufchia et al. based on the VigiBase reports, there were only 94 reports of bradycardia among 2,603 patients who received Remdesivir (3.6%), and 17% developed fatal bradycardia. (Touafchia et al., 2021). The low overall incidence of bradycardia in this study could be due to the indirect investigation of Remdesivir complication reports possibly leading to underestimation and selection bias. (Pallotto et al., 2021b; Touafchia et al., 2021). The present study has a large sample size estimatedprioribased on the literature review, and the patients were selected randomly to limit the selection bias and maintain the external validity. Also, the prospective design of this study maintained the direct evaluation of patients in a real-life setting. These, along with controlling potential cofounders by takingcomplete history and concise methods formeasurements to maintain the internal validity, are the strong points of the present study.

The results of the present study showed that the incidence of bradycardia increased over continuous exposure to Remdesivir. The highest incidence of bradycardia occurred within the five sessions of drug administration (21%), with the most HR reduction compared to baseline. Accordingly, no case of bradycardia was observed after the first session of Remdesivir administration. The mean HR decreased significantly with each drug administration. This can be explained by the accumulative toxicity effect of Remdesivir observed by Choi et al. (Choi et al., 2020; Kumar et al., 2021a) who revealed that Remdesivir cell toxicity increases over time. They observed that the viability of cardiomyocytes considerably decreased by a longer treatment with Remdesivir (48 vs. 24 h). (Choi et al., 2020). This observation is also consistent with the study of Bistrovic et al., who found the frequency of bradycardia consistently increased with every further dose of Remdesivir administration, indicating the causal relation between Remdesivir and bradycardia. (Bistrovic et al., 2022). They also observed that the increased level of Remdesivir above the estimated level of peak plasma concentration was potentially associated with QT interval prolongation. The spontaneous beating was almost completely blocked at higher doses of Remdesivir in their experiment. (Choi et al., 2020). Jung et al. also found that the risk of developing serious cardiac complications increases with drug accumulation or overdose. (Jung et al., 2022). They suggested ECG monitoring during Remdesivir administration, especially for severe COVID-19 infection cases as well as those with structural heart diseases. (Choi et al., 2020; Nabati and Parsaee, 2022). we found that cardiovascular disease and Tocilizumab administration associated with bradycardia in multivariable regression model but it was not confirmed in the multivariable model.

In the present study, the mean QTc interval duration shortened significantly after the fifth dose of Remdesivir compared to baseline. This was in contrast with the hypothesis about Remdesivir induced QTc prolongation. Remdesivir has the potential to inhibit the potassium channel encoded by the human ether-a-go-go gene (hERG) and prolongs the ventricular repolarization, causing QT prolongation and torsades de pointes. (Haghjoo et al., 2021; Michaud et al., 2021; Touafchia et al., 2021).In contrast to the present results, the study of Haghjoo et al. on 67 COVID-19 patients treated with Remdesivir showed a significant increase in QTc interval duration but no arrhythmic event such as torsades de pointes (Tdp) was observed. Their only case with critical QTc prolongation was under treatment with Azithromycin and Remdesivir. (Haghjoo et al., 2021). Gupta et al. (Gupta et al., 2020) reported a case with COVID-19 who developed critical QTc prolongation on the third dose of Remdesivir (>555). However, this patient had received Azithromycin as well, which is known to cause QTc prolongation. (Gupta et al., 2020). In a prospective study, Bistrovic et al. investigated 14 patients with COVID-19 infection and found no significant difference in QTc interval and HR after Remdesivir administration. (Bistrovic and Lucijanic, 2021). Even though the mean QTc duration was reduced in the present study, three patients (1.6%) developed extreme QTc prolongation (QTc>500) after Remdesivir administration. It is noteworthy that none of these patients had a history of prior cardiovascular structural diseases and other comorbidities and risk factors for QTc prolongation or a clinically severe COVID-19 infection. They all remained asymptomatic, and none developed consequent arrhythmia related to QTc prolongation. It appears that Remdesivir has a low potential risk of inducing torsades de pointes, as no case of this and other arrhythmias related to QTc prolongation were observed in this neither study nor previous studies. (Gupta et al., 2020; Haghjoo et al., 2021).

While no association was found between COVID-19 severity indicators and bradycardia, previous studies have shown that SARS-CoV-2 can itself induce bradycardia and arrhythmias in severely infected patients. (Oliva et al., 2021). One possible mechanism for this clinical observation is the cardiotoxicity caused by the inflammation and cytokine release during COVID-19 infection, which may increase the vagal tonicity in the heart. Interleukin 6 (IL6), as an important component of cytokine storm, can increase vagal tonicity. Other mechanisms include the impairment of sinus node normal activity due to direct viral inhibition and defects in the autonomic system function due to direct SARS-CoV-2 toxic effects on the nervous system. (Ye et al., 2018; Hu et al., 2020; Oliva et al., 2021). According to these potential mechanisms, the bradycardia development in the context of severe COVID-19 infection regardless of Remdesivir treatment, may be suggestive of the unfavorable infection course as was observed in Kumar et al. study in which developing bradycardia was associated with a higher mortality rate (OR = 6.59). (Kumar et al., 2021b). Nevertheless, the CRP levels were not suggestive of developing bradycardia in patients in the present study (*p*-value = .41). All studied patients had a favorable prognosis regardless of developing bradycardia and no case of mortality or intubation during hospitalization was observed. The reason could be that most patients in this study had less severe COVID-19 infections and received Remdesivir in an outpatient setting. 19.

Two recent studies revealed that the possibility of developing bradycardia is even higher in less severe COVID-19 cases. (Brunetti et al., 2021; Bistrovic et al., 2022). In a study on 52 patients, The highest HR reduction after Remdesivir treatment was observed in patients with a less clinically severe COVID-19 infection. No association was observed between age, underlying cardiovascular diseases, drugs, and other comorbidities with HR reduction in their multivariate logistic regression analysis. The only significant correlation of bradycardia was observed in less severe COVID-19 infection cases. (Brunetti et al., 2021).

This was similar to the result of this study as to no association between age, comorbidities and risk factors, drug history and developing bradycardia in the multivariate regression model observed. It is noteworthy that in their study, 76% of the subjects were older than 50, 53% had an underlying cardiovascular disease, and 77% had severe COVID-19 presentation. These observations suggest the absence of contraindication when administrating Remdesivir to even critical patients and those with cardiovascular diseases and risk factors despite what was generally hypothesized. (Brunetti et al., 2021). Bistrovic et al. conducted a retrospective investigation on 455 patients who received Remdesivir for the COVID-19 infection and found that the prevalence of bradycardia was significantly higher among survived patients compared to those who died (19% vs. 7%). They observed that developing bradycardia caused by Remdesivir had a significant relationship with a favorable disease course and prognosis. (Bistrovic et al., 2022). The reason may be the intensified sympathetic-adrenergic simulation in patients with severe infections and respiratory failure or that the higher concentrations of Remdesivir metabolites lead to higher simultaneous antiviral and chronotropic effects. So, developing bradycardia following Remdesivir administration should encourage clinicians to continue rather than discontinue the treatment. However, close monitoring is suggested, especially for patients with comorbidities who need synchronous medications for their underlying clinical conditions. (Bistrovic et al., 2022; Lucijanic and Bistrovic, 2022).

In the present study, almost all cases with bradycardia were asymptomatic, and for all patients, sinus bradycardia was transient and returned to normal HR after Remdesivir discontinuation. This is consistent with other case reports about Remdesivir-induced bradycardia being a transitory phenomenon. Developing bradycardia did not affect the clinical outcome of patients in the present study and did not impede the continued drug courses. Only one patient experienced presyncope symptoms at the fifth Remdesivir dose with an extreme decrease in the HR to 25bpm. The drug administration stopped, and the patient received Atropine. The HR returned to normal, and the patient could receive Remdesivir in the following days. None of the studied patients developed an arrhythmia, including atrial fibrillation and cardiac arrest. This finding may be due to the scarcity of these complications, and further investigation of these complications with larger sample sizes is required.

## 5 Limitations

The sample size was small for rare cardiac complications of Remdesivir, such as atrial fibrillation, cardiac arrest, and other rarely reported cardiac arrhythmias.

## 6 Conclusion

Sinus bradycardia is a prevalent adverse cardiac effect of Remdesivir. It is recommended that all COVID-19 patients receiving Remdesivir, be evaluated for heart rate based on examination; and in the case of bradyarrhythmia, cardiac monitoring should be performed during administration to prevent adverse events.

## Data Availability

The raw data supporting the conclusion of this article will be made available by the authors, without undue reservation.
